# A refined prediction model for survival in hepatocellular carcinoma patients treated with transarterial chemoembolization

**DOI:** 10.3389/fonc.2024.1354964

**Published:** 2024-03-28

**Authors:** Hae Lim Lee, Seok Hwan Kim, Hee Yeon Kim, Sung Won Lee, Myeong Jun Song

**Affiliations:** ^1^ Department of Internal Medicine, College of Medicine, The Catholic University of Korea, Seoul, Republic of Korea; ^2^ Korean Liver Cancer Study Group, Seoul, Republic of Korea; ^3^ Ministry of Health and Welfare, Korea Central Cancer Registry, Goyang-si, Gyeonggi-do, Republic of Korea

**Keywords:** hepatocellular carcinoma, overall survival, prediction, transarterial chemoembolization, treatment

## Abstract

**Background/Aims:**

Transarterial chemoembolization (TACE) is widely performed as a major treatment for hepatocellular carcinoma (HCC) patients, and there is a need to stratify patients for whom the most benefit from the treatment. This study aimed to develop a refined prediction model for overall survival (OS) in patients undergoing TACE as a first-line treatment in a large cohort and validate its performance.

**Methods:**

A total of 2,632 patients with HCC of Barcelona Clinic Liver Cancer stage A or B who underwent TACE between 2008 and 2017 were enrolled. The patients were randomly assigned to a training cohort (n = 1,304) or a validation cohort (n = 1,328). Independent predictors of OS were used to develop a prediction model.

**Results:**

The median age of patients in the entire cohort was 63 years, with the majority having hepatitis B virus (56.6%) and being classified as Child-Pugh class A (82.4%). We developed a new prognostic model, called the TACE-prognostic (TP) score, based on tumor burden (sum of the largest tumor diameter and tumor number), alpha-fetoprotein, and Albumin-Bilirubin grade. Patients were classified into five risk groups according to TP scores, with median survival significantly differentiated in both training and validation cohorts (*P* < 0.001). The new model consistently outperformed other currently available models in both the training and validation cohorts.

**Conclusion:**

This newly developed TP scoring system has the potential to be a useful tool in identifying ideal candidates of TACE and predicting OS with favorable performance and discrimination. However, further external validation is needed to confirm its effectiveness.

## Introduction

The Barcelona Clinic Liver Cancer (BCLC) strategy for hepatocellular carcinoma (HCC), which is globally recognized and was recently updated in 2022, offers comprehensive treatment recommendations for different disease stages (1). However, determining the most suitable treatment for HCC is a complex process given the multitude of options available, such as surgery, radiofrequency ablation, transarterial chemoembolization (TACE), radiotherapy, and systemic chemotherapy. Consequently, personalized decisions with a multidisciplinary approach are applied to each patient, taking into account the treatment guidelines.

A considerable number of HCC patients are in an unresectable state at diagnosis. Curative treatments such as surgical resection and radiofrequency ablation (RFA) are limited to 30% of patients, and the most common treatment modality is TACE (2–4). Patients with an intermediate stage of HCC (BCLC B) are generally recommended for TACE. However, patients with early-stage HCC and, less commonly, those with advanced-stage disease, are also considered candidates depending on factors such as tumor location, comorbid diseases, or other relevant aspects; these patients form a proportion of approximately 40% of TACE cases (4). This approach aligns with the stage migration strategy recommended by international guidelines (1, 4–6). As a result, patients who undergo TACE exhibit a wide spectrum of responses in terms of liver function and tumor burden, which has contributed to the heterogeneity in overall survival (OS) in several studies. While the survival benefit of TACE over supportive care has been demonstrated (7, 8), the subgroup of patients who would benefit most from TACE compared to other treatments has not yet been identified.

Several prognostic models have been developed to predict OS in patients with HCC who undergo TACE, including the hepatoma arterial-embolization prognostic (HAP), modified hepatoma arterial-embolization prognostic (mHAP-II), BCLC subclassification, and Six-and-Twelve criteria (9–12). However, these models were developed using relatively small sample sizes and need validation in larger cohorts (13). Furthermore, the emergence of newer systemic chemotherapies, such as tyrosine kinase inhibitors or immune checkpoint inhibitors, has expanded treatment indications for systemic chemotherapy, leading to improved OS, even in patients with intermediate stages of HCC (14). Therefore, it is essential to develop an accurate prognostic model to select ideal candidates for TACE.

The aim of this study was to refine a prognostic model for patients who receive TACE in a large cohort. The objectives of this study were as follows: 1) identify predictors of survival in a cohort of HCC patients undergoing TACE for unresectable HCC, 2) develop and validate a simple scoring system based on the identified predictors, and 3) compare the new scoring system with previously reported models to assess its effectiveness in identifying ideal candidates for TACE.

## Methods

### Patients

**Figure 1:**
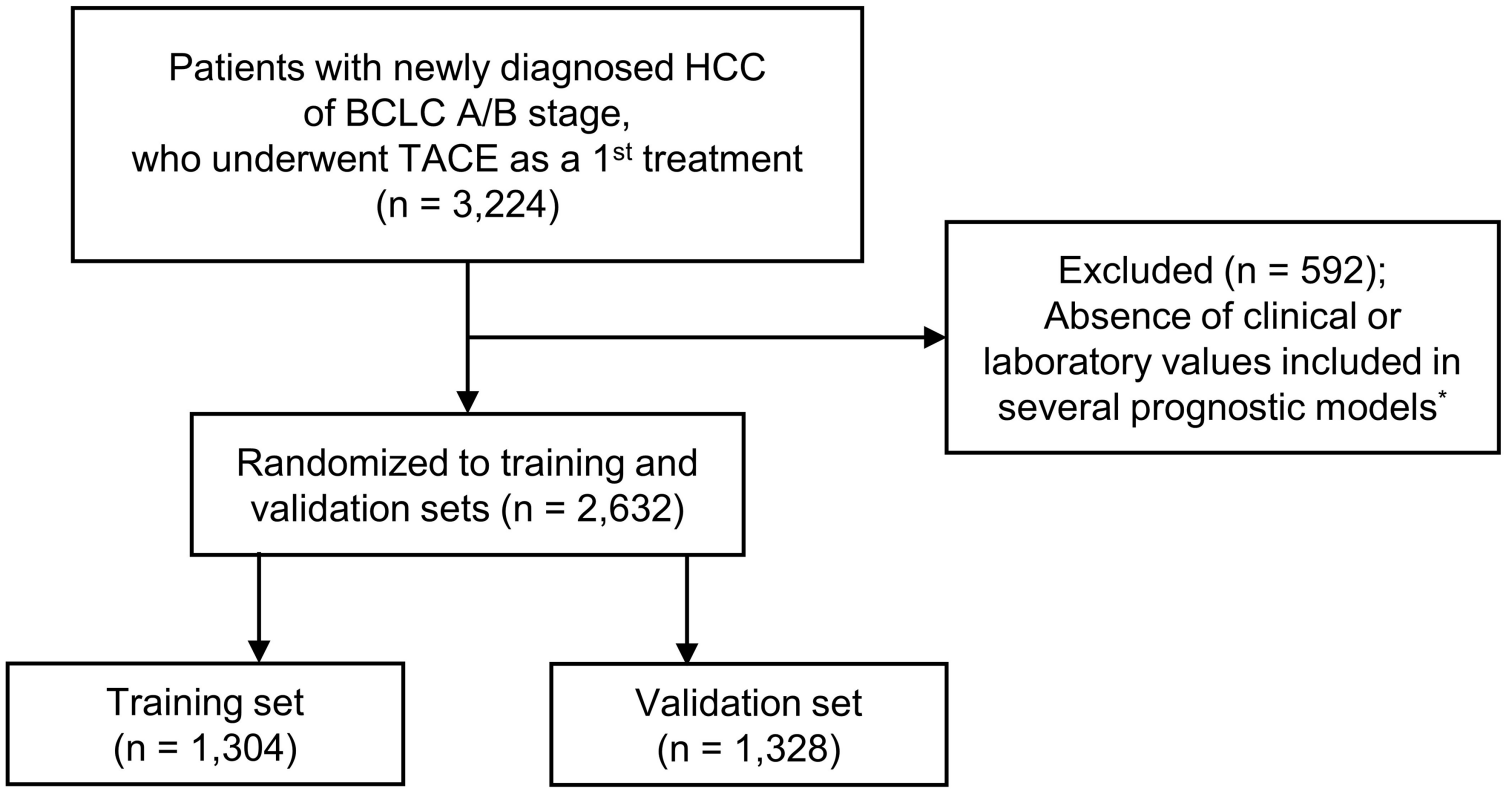
Flow chart of patient enrollment. ^*^Prognostic models: the Child-Turcotte-Pugh score, Model for End-Stage Liver Disease score, Albumin-Bilirubin grade, Up-To-Seven criteria, Hepatoma Arterial-embolization Prognostic (HAP), modified HAP-II, BCLC classification. BCLC, Barcelona Clinic Liver Cancer; HCC, hepatocellular carcinoma; TACE, transarterial chemoembolization.

Patient data were provided by the Korean Liver Cancer Association (KLCA). Since 1980, the Korean Central Cancer Registry (KCCR) has been maintained by the South Korean Ministry of Health and Welfare as a database of all newly diagnosed cancer cases. The KLCA extracted and registered HCC cohort data randomly from the KCCR database. A total of 3,224 patients with newly diagnosed HCC of BCLC stage B or A who underwent TACE as their first treatment between 2008 and 2017 were reviewed for study eligibility. While patients with BCLC stage A are generally indicated for curative treatment, a significant proportion of patients ultimately opted for TACE as the first-line treatment, following the stage migration strategy outlined in international guidelines (1, 4, 5). Patients without clinical or laboratory values included in prognostic models, such as Child-Turcotte-Pugh (CTP) or Model for End-stage Liver Disease (MELD) scores, Albumin-Bilirubin (ALBI) grade, Up-To-Seven criteria, HAP, mHAP-II, or BCLC classification, were excluded (n = 592). Ultimately, 2,632 patients were enrolled and randomly assigned to either a training (n = 1,304) or validation (n = 1,328) cohort (**Figure 1**). Following BCLC guidelines, there were no patients with decreased liver function of CTP class C, major portal vein tumor invasion, or extrahepatic metastasis. This study was approved by the Institutional Ethics Review Board of The Catholic University of Korea (DC21ZISI0087) and conducted in accordance with the Declaration of Helsinki. The need for informed consent was waived by the Institutional Ethics Review Board.

### Endpoint and diagnosis

The primary outcome was OS. Follow-up duration was from the date of HCC diagnosis until December 2020 or the date of death.

The diagnosis of HCC in this study followed the guidelines of the Korean Liver Cancer Study Group and the National Cancer Center of Korea (5). The diagnostic criteria were as follows: (1) pathological diagnosis, and (2) diagnosis based on one or two imaging modalities showing liver nodule ≥ 1cm in high-risk patients, such as those with hepatitis B virus (HBV) or hepatitis C virus (HCV) infection or liver cirrhosis (LC). The imaging modalities were evaluated for specific characteristics indicative of HCC, including hypervascularity in the arterial phase and washout in the portal or delayed phase of dynamic computed tomography, dynamic magnetic resonance imaging (MRI), or gadolinium-ethoxybenzyl-diethylenetriamine pentaacetic acid–enhanced MRI.

### Clinical data and treatment procedures

Baseline clinical data were collected at or around the date of HCC diagnosis, prior to initiating treatment. The data included age, sex, etiology of HCC, tumor characteristics, laboratory data including liver function tests, serum tumor markers of alpha-fetoprotein (AFP) and protein induced by vitamin K absence or antagonist-II, and performance status. The MELD score was calculated using the following formula: 3.78 × ln (bilirubin, mg/d) + 11.2 × ln (prothrombin time, INR) + 9.57 × ln (creatinine, mg/dL) + 6.43. The ALBI score was calculated using the following formula: [log10 (bilirubin, μmol/L) × 0.66] + (albumin, g/L × −0.085). The ALBI grades were defined as follows: Grade 1, ≤ −2.60; Grade 2, > −2.60 and ≤ −1.39; Grade 3, > −1.39 (15).

TACE was predominantly conducted using doxorubincin or cisplatin, with occasional use of epirubicin, idarubicin, or other chemotherapeutic agents, mixed with lipiodol. In some cases, a combination of these agents was administered in accordance with the practices of individual centers. The delivery of embolic agents following the infusion of chemoemulsion was guided by the tumor-feeding artery and location of the microcatheter and administered as selectively as possible (16). Following the initial treatment with TACE, approximately 70% of the enrolled patients underwent additional TACE procedure to achieve complete tumor necrosis, while others received curative treatment such as RFA or resection, radiotherapy, systemic chemotherapy, or supportive care.

### Statistical analysis

Categorial variables were expressed as number (%) and continuous variables were expressed as mean ± SD or median (interquartile range). Categorical variables were compared using the Chi-square test or Fisher’s exact test, while continuous variables were compared using Student’s t test or non-parametric Mann-Whitney U test, depending on the appropriateness of the test for each variable. OS and the comparison of patient groups categorized according to each prognostic model were assessed using Kaplan-Meier curves with log-rank tests in both the training and validation sets. Independent association between OS and clinical factors (sex, age, etiology, largest tumor size, tumor number, AFP, ALBI grade, prothrombin time, alanine aminotransferase (ALT), platelet) were assessed by conducting univariable and multivariable analyses using Cox proportional-hazard model. The proportional hazards assumption was evaluated with the use of Schoenfeld residuals.

We developed two prediction models incorporating independent prognostic factors for OS in HCC patients treated with TACE. Model 1 was developed using the coefficients derived from each factor in the multivariable analysis. Model 2 was specifically designed to simplify calculation for the prediction model. The scores assigned to each factor in Model 2 were determined based on the HR observed in the multivariable analysis. The predictability of these models was evaluated by comparing the C-index and time-dependent area under the receiver operating characteristic (AUROC) curve with those of previous models. SAS version 9.4 statistical software (SAS Institute, Inc.; Cary, NC, United States) and R version 3.3.2 were used in analysis of all statistical values. *P*-values were considered significant at ≤ 0.05.

## Results

### Baseline characteristics

The 2,632 patients included in this study were randomly divided into the training (n = 1,304) and validation (n = 1,328) cohorts. The median age of the patients was 63 years (55–71), and most patients (78.1%) were male. The most common etiology of HCC was HBV (56.6%), followed by HCV (14.1%) and alcohol consumption (13.5%). Most patients (82.4%) were classified as CTP class A, and the median MELD score was 8 (7–10). The most frequent ALBI grade was 2 (50.8%), followed by 1 (44.1%) and 3 (5.1%). Single tumors were present in 50.2% of patients, with tumors of 5cm or less observed in 79.4% of patients. Patients with BCLC stage A constituted 66.2% of the cohort, while those with BCLC Stage B comprised 33.9%. The baseline characteristics of the patients are summarized in **Table 1**.

**Table 1:** Baseline characteristics.

	Entire cohort (n = 2,632)	Training cohort (n = 1,304)	Validation cohort (n = 1,328)	*P*
Sex				0.364
Male	2056 (78.1)	1009 (77.4)	1047 (78.8)	
Female	576 (21.9)	295 (22.6)	281 (21.2)	
Age (years)	63 (55–71)	62 (54–70)	63 (56–72)	0.014
Etiology				0.973
HBV	1489 (56.6)	734 (56.3)	755 (57.0)	
HCV	371 (14.1)	182 (14.0)	189 (14.2)	
Alcohol	356 (13.5)	179 (13.7)	177 (13.3)	
others	416 (15.8)	209 (16.0)	207 (15.6)	
Tumor number				0.814
Single	1322 (50.2)	645 (49.5)	677 (51.0)	
Multiple	1310 (49.8)	659 (50.5)	651 (49.0)	
Largest tumor diameter, cm				0.610
≤ 5	2089 (79.4)	1045 (80.1)	1044 (78.6)	
> 5, ≤ 10	455 (17.3)	216 (16.6)	239 (18)	
> 10	88 (3.3)	43 (3.3)	45 (3.4)	
BCLC staging				0.777
A	1741 (66.2)	866 (66.4)	875 (65.9)	
B	891 (33.9)	438 (33.6)	453 (34.1)	
CTP score				0.027
5, 6	2169 (82.4)	1053 (80.8)	1116 (84.0)	
7, 8, 9	463 (17.6)	251 (19.3)	212 (16.0)	
MELD	8 (7–10)	8 (7–10)	8 (7–10)	0.368
ALBI grade				0.187
1	1161 (44.1)	553 (42.4)	608 (45.8)	
2	1336 (50.8)	679 (52.1)	657 (49.5)	
3	135 (5.1)	72 (5.5)	63 (4.7)	
Albumin (g/dl)	3.9 (3.4–4.2)	3.8 (3.4–4.2)	3.9 (3.4–4.2)	0.031
Bilirubin (mg/dl)	0.9 (0.6–1.3)	1.0 (0.6–1.3)	0.9 (0.6–1.3)	0.275
Prothrombin time (INR)	1.1 (1.0–1.2)	1.1 (1.0–1.2)	1.1 (1.0–1.2)	0.275
ALT (IU/mL)	34 (22–52)	34 (21–52)	34 (22–51)	0.603
Platelet (×10^9^/L)	122 (85–169)	118 (83–163)	125 (87–177)	0.014
AFP (ng/mL)	18 (6–166)	19 (6–170)	18 (6–162)	0.144
AFP (ng/mL)				0.081
≤ 200	1161 (44.1)	553 (42.4)	608 (45.8)	
> 200	1471 (55.9)	751 (57.6)	720 (54.2)	
PIVKA II (mAU/mL)^*^	69 (25–394)	61 (24–372)	76 (26–465)	0.236
Follow-up duration (month)	43.7 (21.3–73.0)	42.7 (20.8–70.0)	44.7 (21.8–75.1)	0.018

Data are expressed as the number (%) for categorical variables, and mean ± SD or median (interquartile range) for continuous variables.

^*^ There were missing values for 866 patients.

AFP, alpha-fetoprotein; ALBI, albumin-bilirubin; ALT, alanine aminotransferase; BCLC, Barcelona clinic liver cancer; CTP, Child-Turcotte-Pugh; HBV, hepatitis B virus; HCV, hepatitis C virus; MELD, Model for End-stage Liver Disease; PIVKA-II, protein induced by vitamin K absence or antagonist-II.

### Overall survival

**Figure 2:**
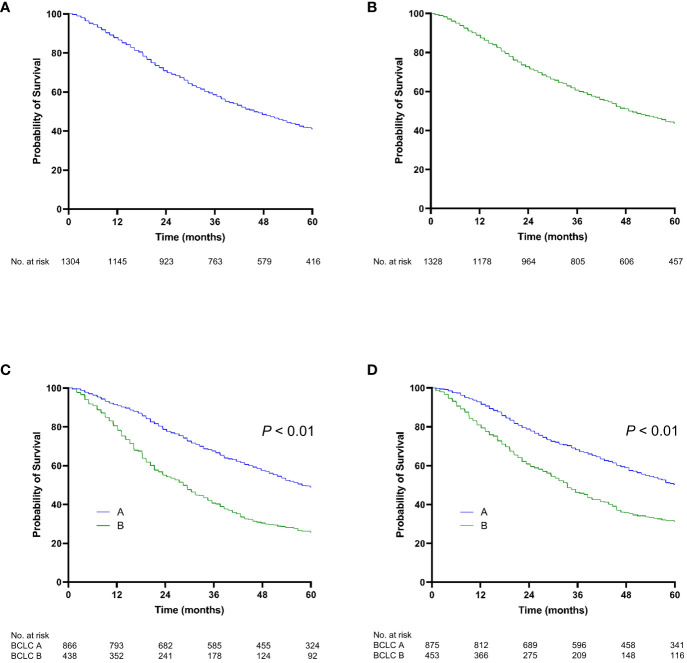
Kaplan-Meier curve of overall survival. **(A)** The training cohort; **(B)** the validation cohort; **(C)** stratified by BCLC stage for the training cohort; **(D)** stratified by BCLC stage for the validation cohort.

The median follow-up was 42.7 months (20.8–70.0) in the training set and 44.7 months (21.8–75.1) in the validation set. During follow-up, 890 (68.3%) and 877 (66.0%) patients in the training and validation sets died, respectively. The median survival of the entire cohort was 45.6 months (22.3–77.1); the 2-year, 5-year, and 10-year survival rates were 71.6%, 42.3%, and 23.4%, respectively. There was no significant difference in the median OS between the training and validation cohorts (*P* = 0.198) (**Figures 2A, B**).

In the training set, the OS rates at 2 and 5 years were 78.8% and 48.9%, respectively, for patients with BCLC stage A and 55.0% and 25.6% for those with BCLC stage B. In the validation set, the OS rates at 2 and 5 years were 78.7% and 50.1% for patients with BCLC stage A and 60.7% and 31.2% for those with BCLC stage B (**Figures 2C, D**). Liver function grades, including the ALBI grade and CTP class, significantly stratified OS (*P* < 0.001) (**Figures S1A–D**). Moreover, various tumor-associated grading models, such as Up-To-Seven, HAP, and mHAP-II, significantly enabled stratification of OS (*P* < 0.001) (**Figures S2A–F**).

### Univariable and multivariable analyses of overall survival

Factors associated with OS were evaluated in the training cohort (**Table 2**). In the univariable analysis, several factors were significantly associated with OS, including age, etiology, largest tumor size, tumor number, AFP level, and ALBI grade. In the multivariable analysis, age (adjusted HR [aHR] 1.0, 95% confidence interval [CI] 1.0–1.0, *P* < 0.001), etiology of HBV (aHR 0.8, 95% CI 0.8–0.9, *P* = 0.001), largest tumor size (aHR 1.5, 95% CI 1.3–1.7, *P* < 0.001 for tumor size > 5 cm to ≤ 10 cm; aHR 3.2, 95% CI 2.5–4.0, *P* < 0.001 for tumor size > 10 cm; compared with tumor size ≤ 5 cm as the reference), multiple tumor (aHR 1.3, 95% CI 1.2–1.5, *P* < 0.001), AFP > 200 ng/mL (aHR 1.4, 95% CI 1.2–1.5, *P* < 0.001), and ALBI grade 2/3 (aHR 1.7; 95% CI 1.6–1.9; *P* < 0.001) remained as independent predictors of OS.

**Table 2:** Predictors of overall survival.

Risk factors	Univariable		Multivariable	
	Adjusted HR (95% CI)	*P*	Adjusted HR (95% CI)	*P*
Sex (male)		0.850		
Age	1.0 (1.0, 1.0)	< 0.001	1.0 (1.0, 1.0)	< 0.001
Etiology				
HBV	0.7 (0.6, 0.7)	< 0.001	0.8 (0.8, 0.9)	0.001
Non-HBV	reference		reference	
Largest tumor size, cm				
≤ 5	reference		reference	
> 5, ≤ 10	1.6 (1.5, 1.8)	< 0.001	1.5 (1.3, 1.7)	< 0.001
> 10	3.6 (2.8, 4.5)	< 0.001	3.2 (2.5, 4.0)	< 0.001
Tumor number				
Single	reference		reference	
multiple	1.3 (1.2, 1.4)	< 0.001	1.3 (1.2, 1.5)	< 0.001
AFP (ng/mL)				
≤ 200	reference		reference	
> 200	1.5 (1.3, 1.6)	< 0.001	1.4 (1.2, 1.5)	< 0.001
ALBI grade				
1	reference		reference	
2/3	1.7 (1.5, 1.9)	< 0.001	1.7 (1.6, 1.9)	< 0.001
Prothrombin time, INR		0.521		
ALT (IU/mL)		0.721		
Platelet (×10^9^/L)		0.327		

AFP, alpha-fetoprotein; ALBI, albumin-bilirubin; ALT, alanine aminotransferase; HR, hazard ratio; HBV, hepatitis B virus.

### Development of the prognostic model

We developed two prediction models using the four independent predictive factors for OS: the largest tumor size, tumor number, AFP level, and ALBI grade.

Model 1 was formulated with the following equation: 1.1 × the largest tumor size + 1.6 × tumor number + 2.2 × AFP + 4.3 × ALBI grade (1 and 2/3). Tumor size, tumor number, and AFP level were considered as continuous variables in this model.

Model 2 was formulated with the following equation: the sum of the largest tumor size and tumor number + AFP level + ALBI grade. AFP level (≥ 200 ng/mL and < 200 ng/mL) and ALBI grade (1 and 2/3) were used as categorical factors. The sum of the largest tumor size and tumor number was treated as a continuous factor in the model. The cut-off values for the sum of the largest tumor size and tumor number were determined as the first and third quartiles of the corresponding values in the patients from the training cohort. The scores of Model 2 ranged from 1 to 3 for tumor burden (sum of tumor size and number) and from 0 to 1 for AFP and ALBI grades (**Table 3**).

**Table 3:** TACE-prognostic (TP) score (Model 2).

	Point
Tumor size and number^*^	
≤ 5	1
> 5, ≤ 10	2
> 10	3
AFP	
< 200	0
≥ 200	1
ALBI grade	
1	0
2,3	1

^*^The sum of the largest tumor size and tumor number.

### Predictive performance

**Figure 3:**
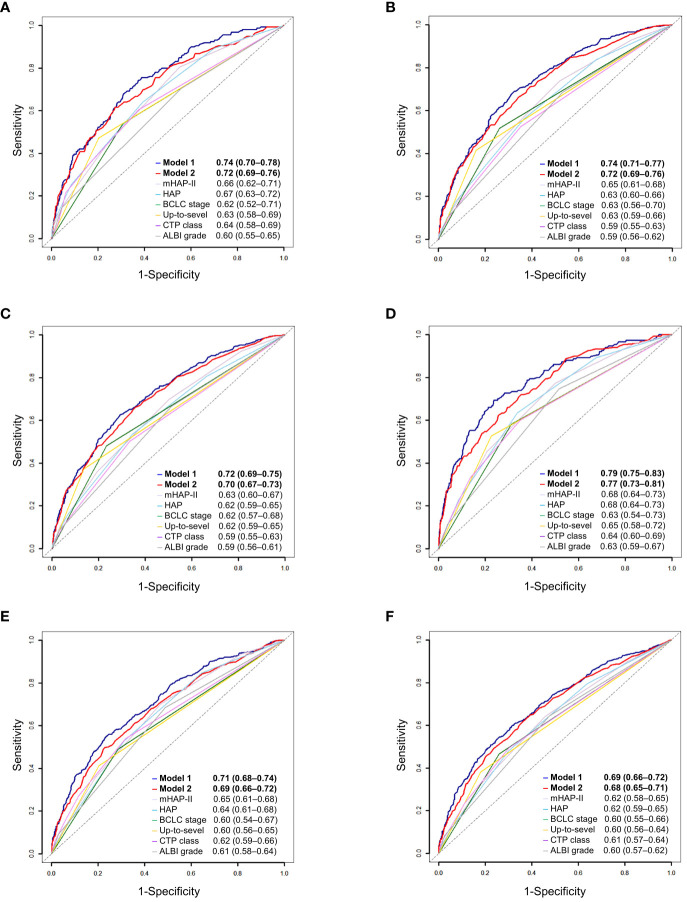
Time-dependent area under the receiver-operating characteristics (AUROC) of prediction models for patients with hepatocellular carcinoma. **(A)** 1-year, **(B)** 2-year, and **(C)** 3-year AUROCs of Model 1. **(D)** 1-year, **(E)** 2-year, and **(F)** 3-year AUROCs of Model 2.

**Figure 4:**
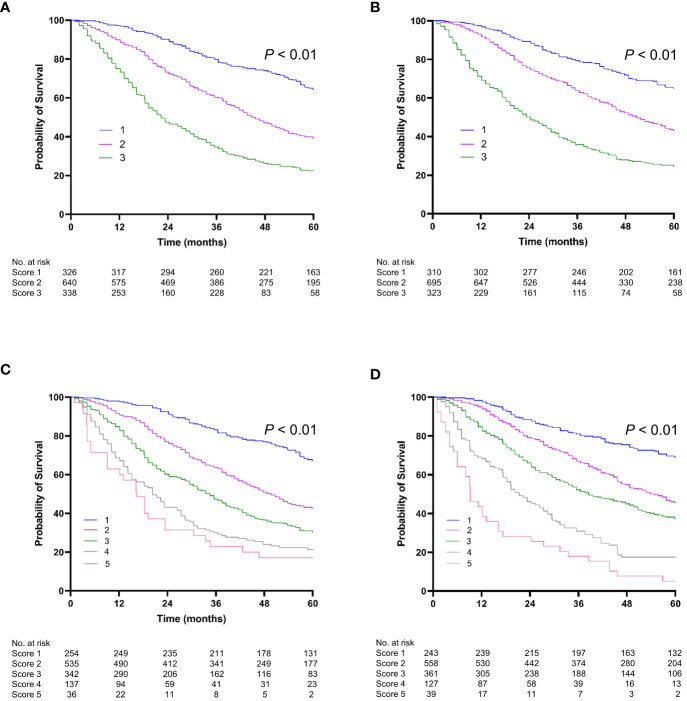
Kaplan-Meier curve of overall survival (OS). Stratified by Model 1 **(A)** for the training cohort; **(B)** for the validation cohort. Stratified by Model 2 (TP score) **(C)** for the training cohort; **(D)** for the validation cohort.

We compared the performance of the two new prediction models (Model 1 and Model 2) with other existing models and found that the two new models had the highest 1-year, 2-year, and 3-year AUROCs and C-indices in both the training and validation cohorts (**Table 4** and **Figure 3**). In Model 1, patients were categorized into three groups based on the first and third quartiles of the risk score distribution within the cohort. The OS rates were significantly different among these patient groups in both the training and validation cohorts (*P* < 0.001) (**Figures 4A, B**), with a median OS of 126.8 (111.6–not determined), 50.8 (48.7–53.8), and 12.6 (12.2–14.1) months, respectively, in the entire cohort. In Model 2, the OS rate also exhibited significant differences by score in both the training and validation cohorts (*P* < 0.001) (**Figures 4C, D**). The median OS was 102.5 (93.4–109.6), 51.8 (47.7–55.8), 35.5 (33.5–39.6), 20.8 (18.3–25.4), and 12.2 (9.1–18.3) months for scores ranging from 1 to 5, respectively, in the entire cohort. While Model 1 exhibited slightly higher predictive performance compared with Model 2 without statistical significance for time-dependent AUROCs (*P* > 0.05 at all time points), we prioritized Model 2 due to its superior performance over previous models and its ease of calculation at the bedside or in outpatient clinics. Consequently, Model 2 was ultimately selected as our new prediction model, called TACE-prognostic (TP) scores.

**Table 4:** Comparison of the predictive performance of the models.

Cohort	Models	C-index (95% CI)
**Training**	**Model 1**	0.67 (0.66 - 0.68)
	**Model 2**	0.65 (0.64 - 0.66)
	mHAP-II	0.60 (0.59 - 0.61)
	HAP	0.59 (0.58 - 0.60)
	BCLC stage	0.59 (0.58 - 0.60)
	Up-To-Seven	0.58 (0.57 - 0.59)
	CTP class	0.57 (0.56 - 0.58)
	ALBI grade	0.57 (0.56 - 0.58)
**Validation**	**Model 1**	0.65 (0.64 - 0.66)
	**Model 2**	0.64 (0.63 - 0.65)
	mHAP-II	0.60 (0.59 - 0.61)
	HAP	0.59 (0.59 - 0.61)
	BCLC stage	0.57 (0.56 - 0.58)
	Up-To-Seven	0.57 (0.56 - 0.58)
	CTP class	0.58 (0.58 - 0.59)
	ALBI grade	0.58 (0.57 - 0.59)

ALBI, Albumin-Bilirubin; BCLC, Barcelona clinic liver cancer; AUROC, area under receiver operating characteristic curve; CTP, Child-Turcotte-Pugh; HAP, hepatoma arterial-embolization prognostic; mHAP-II, modified hepatoma arterial-embolization prognostic II.

## Discussion

While several scoring systems have been developed to predict outcomes in patients undergoing TACE for HCC, there is no consensus on which is the best. In this study, we developed a new prognostic model for HCC in a large cohort and compared it with previous models. Our results demonstrated the improved predictive power of the new model. The new TP score consists of tumor size and number, AFP level, and ALBI grade, with the latter two factors newly incorporated into the 2022 BCLC staging system to stratify HCC patients (1). The TP score effectively stratified patients into different OS rates based on their scores. Specifically, patients with a TP score of 5 experienced a lower median OS compared to the expected survival rates for patients with BCLC stage A and B (1).

The factors included in the new models of this study resemble those in the mHAP-II model. The higher predictability of the new models may be attributed to the inclusion of continuum variables for tumor burden. This was achieved by combining the largest tumor size and tumor number, rather than assessing them independently. Additionally, the inclusion of ALBI grade further enhanced the predictive accuracy of the models. Previous studies have demonstrated that models incorporating a continuum variable derived from the sum of independent factors, such as Up-To-Seven criteria or ALBI grade, tend to exhibit higher predictability (15, 17, 18). This comprehensive approach enables a more accurate assessment of patients who undergo TACE, leading to improved predictive accuracy.

This study highlights the importance of including both factors representing tumor burden and liver function in prognostic models for HCC patients undergoing TACE. By comparing the performance of existing models, we observed that the predictive accuracy of models incorporating both tumor burden and liver function, such as HAP and mHAP-II scores, was superior to those that considered only one of these factors. Models that consider only tumor burden, such as the Milan or Up-To-Seven criteria, or the recently introduced Metroticket 2.0 were originally intended for liver transplantation candidates where liver function is not critical to the treatment (17, 19). The 2022 version of the BCLC classification also underscores the importance of liver function and AFP level, as well as tumor burden, when considering treatment strategies for HCC patients (1). TACE is commonly associated with postembolization syndrome, which can cause abdominal pain, fever, loss of appetite, or liver function deterioration, with an incidence of up to 46% (20). Moreover, over 50% of patients exhibit an increase in AST, ALT, or bilirubin level after TACE; the rate of deterioration in CTP score or ALBI grade is approximately 5–15% after each session (20, 21). TACE carries the risk of worsening liver function in patients who are already in a vulnerable state, potentially leading to a devastating outcome. Therefore, it is reasonable to include liver function measures in the prediction model for TACE.

In this study, the median OS of the patients was 45.6 months (22.3–77.1), which is slightly longer than rates in previous studies that developed the prediction models and is more in line with reported rates of approximately 40 months in well-selected patients who underwent a super-selective approach for TACE (12, 22–25). This suggests that the patients enrolled in this study were ideal candidates for TACE. Despite this, the TP score was effective in discriminating the patients into five risk groups based on OS. Notably, the median OS was not significantly different between patient groups divided by AFP level when patients were in the same category of tumor burden and ALBI grade of 1. However, it is worth noting that among patients with ALBI grade 2 or 3 and the same tumor burden category, there was a significant variation of approximately 10 months in median OS between groups divided by AFP level of 200 ng/mL. As approximately 50% of this study population had liver function of ALBI grade 2 or 3, the AFP level is a non-negligible factor in the TP score as a predictor of OS.

As a measure of liver function, ALBI grade showed higher association with mortality than CTP or MELD scores in this study. ALBI grading was originally developed to predict OS in patients with HCC. Its predictive power has been validated in HCC patients across all stages of BCLC classification, as well as in patients with LC but no HCC (26). The superiority of ALBI grade over the CTP classification was demonstrated by differentiating the OS in patient groups classified by ALBI grade into 1 and 2, even among patients whose CTP class was A (15). Several studies of patients with intermediate-stage HCC treated with TACE have shown that the ALBI grade outperformed CTP score in predicting prognosis (24, 27–29). In this cohort, the mean OS of patients with ALBI grade 1 was more than 13 months longer than that of patients with ALBI grades 2 or 3, when they were in the same category divided by tumor number and size and AFP levels.

According to the 2022 BCLC strategy, patients with BCLC stage B are expected to have a median OS longer than 2.5 years (1). However, the median OS of the patients classified as TP score 5 was 12.2 (9.1–18.3) months, indicating the need for change in the treatment strategy beyond TACE alone for these patients. Tumors that are classified as TP score 5, which can include massive tumors or small tumors with several daughter nodules even within BCLC stage B, may be TACE-unsuitable or TACE-resistant (30). In such cases, repeated TACE can lead to a decline in liver function and poor survival outcomes (31). Additionally, studies have reported that insufficient treatment response to TACE in large tumors may increase the potential for the tumor to evolve into a more aggressive type, such as sarcomatous change or poorly differentiated histology (32). Therefore, the stage migration strategy outlined in international guidelines can be applied to such cases.

This study has several limitations that need to be addressed. First, as patient data were extracted from the KCCR database, information regarding response evaluation for TACE, such as radiological response or time to tumor progression following TACE, was not available. However, evaluating the response to TACE can be challenging, primarily because TACE treatment is frequently combined with other modalities, including radiotherapy, RFA, surgery, and even systemic chemotherapy. In most studies, OS remains the mainstay of prognostic prediction. Second, the study cohort was relatively homogeneous due to the population characteristics of South Korea, where HBV is the most prevalent cause of HCC and the ethnic diversity is limited. Therefore, it is necessary to evaluate the validity of the TP score in other clinical practices.

In conclusion, we developed a straightforward and clinically relevant prognostic model using baseline factors for patients undergoing TACE. We anticipate that TP score could serve as a helpful guide for personalized treatment decision-making. However, further research is needed to prospectively validate our findings in a large cohort.

## Data availability statement

The raw data supporting the conclusions of this article will be made available by the authors, without undue reservation.

## Ethics statement

The studies involving humans were approved by The Institutional Ethics Review Board of The Catholic University of Korea (DC21ZISI0087). The studies were conducted in accordance with the local legislation and institutional requirements. Written informed consent for participation was not required from the participants or the participants’ legal guardians/next of kin in accordance with the national legislation and institutional requirements.

## Author contributions

HL: Conceptualization, Data curation, Formal analysis, Investigation, Methodology, Project administration, Resources, Software, Validation, Visualization, Writing – original draft, Writing – review & editing. MS: Conceptualization, Data curation, Formal analysis, Funding acquisition, Investigation, Methodology, Project administration, Resources, Software, Supervision, Validation, Visualization, Writing – original draft, Writing – review & editing. HK: Methodology, Supervision, Writing – review & editing. SL: Methodology, Supervision, Writing – review & editing. SK: Methodology, Supervision, Writing – review & editing.
