# Supplementary material for: A refined prediction model for survival in hepatocellular carcinoma patients treated with transarterial chemoembolization
Source: Front Oncol. 2024 Mar 28;14:1354964. doi: 10.3389/fonc.2024.1354964 (PMC11007070; doi:10.3389/fonc.2024.1354964)

**Supplementary data**

**Supplementary Figure 1.** Kaplan-Meier curve of overall survival. (A) Stratified by Albumin-Bilirubin (ALBI) grade for the training cohort; (B) stratified by ALBI grade for the validation cohort; (C) stratified by Child-Turcotte-Pugh (CTP) class for the training cohort; (D) stratified by CTP class for the validation cohort.


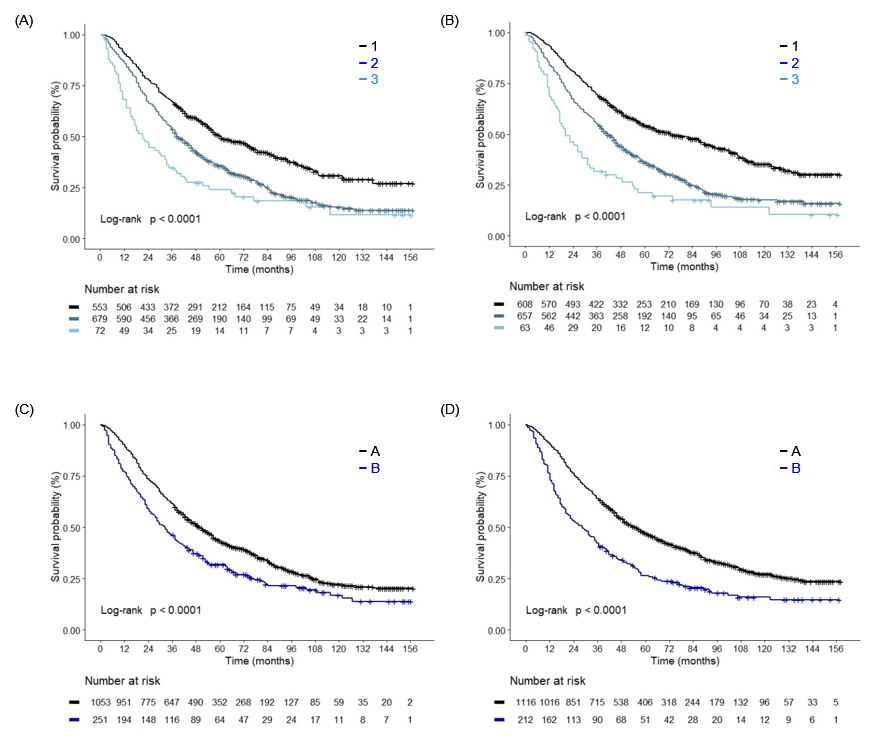


**Supplementary Figure 2.** Kaplan-Meier curve of overall survival. (A) Stratified by Up-To-Seven criteria for the training cohort; (B) stratified by Up-To-Seven criteria for the validation cohort; (C) stratified by hepatoma arterial-embolisation prognostic (HAP) score for the training cohort; (D) stratified by HAP score for the validation cohort; (E) stratified by modified hepatoma arterial-embolisation prognostic (mHAP-II) score for the training cohort; (F) stratified by mHAP-II score for the validation cohort.


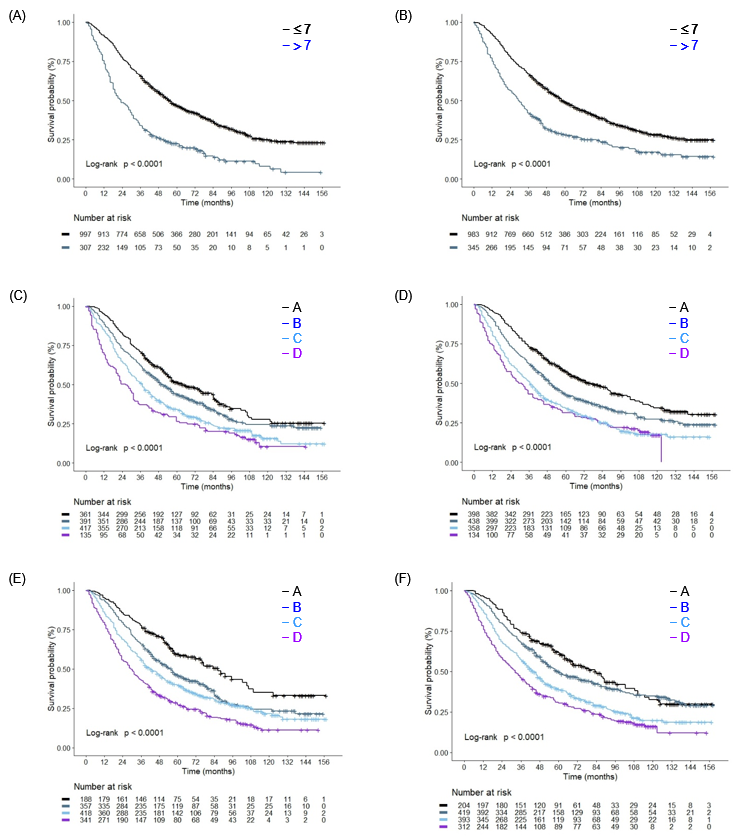

Supplement: Supplementary file 1 [file DataSheet_1.docx]
